# Imaging evaluation in metabolic syndrome: beyond steatosis

**DOI:** 10.1590/0100-3984.2016.49.1e1

**Published:** 2016

**Authors:** Maurício Zapparoli

**Affiliations:** 1 Master, Diagnostic Radiology Residency Program Director and Professor of Radiology at Hospital de Clínicas - Universidade Federal do Paraná (UFPR); Radiologist at Clínica Diagnóstico Avançado por Imagem (DAPI), Curitiba, PR, Brazil. E-mail: mauricioz@dapi.com.br.

Metabolic syndrome is characterized by an association of factors which directly increase the risk of development of cardiovascular atherosclerotic disease and diabetes type 2, such as visceral obesity, dyslipidemia, systemic arterial hypertension, and insulin resistance, with consequential increase in mortality^(1)^. It is estimated that this condition affects 25% of the population worldwide, constituting a relevant public health problem^(2)^. Steatosis is the hepatic manifestation of the metabolic syndrome^(3)^.

In cases where it is related to metabolic syndrome, steatosis is part of the spectrum of nonalcoholic fatty liver disease (NAFLD) - the most frequent type diffuse liver disease. In most cases, it manifests as simple steatosis, but in up to 30% of cases it evolves to steatohepatitis, that may progress to fibrosis and, in 15-20% of cases, to cirrhosis, with increased risk of hepatocarcinoma^(2,4,5)^. Moreover, a direct cause-effect relationship of NAFLD in the pathogenesis of atheromatous disease is probable^(4)^. Early diagnosis and treatment monitoring of NAFLD are therefore of paramount relevance^(3)^.

The Brazilian radiological literature has recently been quite concerned with the relevance of imaging methods in the investigation of liver diseases^(6,11)^. Specifically in cases of steatosis, imaging methods play an important role, as demonstrated by the study developed by Cruz et al.^(12)^, published in the present issue of **Radiologia Brasileira**. In such study, the authors have found that the prevalence of liver steatosis in patients referred to undergo abdominal ultrasonography (US) in Aracaju, SE, Brazil, was 29.1% - similar to the prevalence observed in the international literature -, which brings in an interesting discussion on the matter and demonstrates the value of US for initial, noninvasive detection of NAFLD and qualitative grading of steatosis^(3,4,6,12)^.

More important than quantifying or grading steatosis is to identify patients with NAFLD who evolve or have higher risk to develop steatohepatitis. Liver biopsy is considered the standard of reference for such a purpose, but it is an invasive technique, involving possible complications and subjected to sampling errors^(3,4,13,14)^. Currently, one of the greatest challenges is finding a noninvasive, practical and reproducible method to replace the histological analysis. In this context, magnetic resonance imaging (MRI) is very promising because of its unique ability to extract information from different tissue components and to identify inflammatory activity markers such as iron deposit, edema and fibrosis^(14,15)^.

Even small increases in the iron concentration of the liver seems to be related to insulin resistance, increased risk of steatohepatitis, and development of hepatocarcinoma, stimulating fibrogenesis and carcinogenesis^(16)^. Iron concentration in the liver can easily be determined by MRI with recently developed robust sequences utilizing principles of chemical-shift and T2* relaxometry, for simultaneous and more accurate calculation of the proton density fat fraction (PDFF) and iron concentration in the liver, with correction of bias factors^(13,15)^. Inflammation and necrosis lead to edema, that may be identified by T2-weighted sequences with fat saturation, with potential for semiquantitative grading of necroinflammatory activity based on liver to fat signal intensity ratio^(15)^. MRI elastography is an increasingly used imaging modality that allows for accurate evaluation of hepatic fibrosis, covering the whole liver parenchyma^(17)^.

Metabolic syndrome is of high relevance for radiologists given the capacity of different imaging modalities to demonstrate liver disease in asymptomatic patients, and the study developed by Cruz et al. demonstrates the relevance of US for steatosis screening^(12)^. Due to technological developments MRI has increasingly become important in the supplementary evaluation of patients diagnosed with NAFLD for its capacity to characterize biomarkers that allow selection of patients with higher risk of steatohepatitis and cardiovascular diseases^(15)^. With the validation of more recent and accurate techniques for simultaneous quantitative evaluation of PDFF and iron concentration, and sequences that identify necroinflammatory activity and fibrosis, the method promises to be one-stop-shop in the evaluation of diffuse liver diseases, with a significant impact on the management of these patients.
